# Lessons from Animal Diabetes 8, 24–26 July 2001

**DOI:** 10.1155/EDR.2001.247

**Published:** 2001

**Authors:** Yasunori Kanazawa, Shigehiro Katayama


### Lessons from Animal Diabetes 8
### 24-26 July 2001

YASUNORI KANAZAWA, CHAIRPERSON LAD 8
SHIGEHIRO KATAYAMA, CHAIRPERSON JAADR 15

The 8th International Workshop on Lessons from Animal Diabetes was held July 24-26, 2001 at Nichidai Kaikan in Tokyo, the same venue where the 3rd Workshop took place in 1990. This issue of *Experimental Diabetes Research* is dedicated to providing coverage of the highlights of the lectures and communications presented at the workshop.

The 8th LAD was organized jointly with the 15th annual meeting of Japan Association of Animal Diabetes Research (JAADR). JAADR is a research group initiated by Professor Yoshio Goto who established the famous GK rat model. There were over 200 participants about one half of them from Japan. Thus, the participants of 8th LAD had an opportunity to interact with many Japanese investigators. There was a novel session on "Animal Models of Diabetes Established in Japan," which gave a special Japanese tone to the meeting. Twelve animal models were presented, many already widely known and others new that would get into international use soon.

Among the important features was the 2nd Renold Memorial Lecture presented by Professor Eleazar Shafrir, entitled "Molecular Background of Nutritionally Induced Insulin Resistance and Leading to Type 2 Diabetes – from Animal Models to Humans" (reprinted in this issue). We also had two plenary lectures by Doctor Palle Serup from Gentofte, Denmark on "Pancreatic stem cells and islet cell differentiation" and by Professor Takashi Kadowaki from the University of Tokyo on "Molecular Pathogenesis of Type 2 diabetes in knockout mice models." LAD 8 comprised 4 symposia and 3 workshops in which we discussed the genetics and pathogenic mechanisms relevant to human type 1 and type 2 diabetes. We had 95 presentations, including short communications and posters not only related to pathogenesis of diabetes but also results of detailed mapping of genes, pathogenesis of complications and new therapeutic approaches. Abstracts of these communications appear in this volume. Listening and discussing these presentations, we felt that studies with animal models are opening new fields of diabetes research and significantly contribute to the understanding of pathogenesis of human diabetes and to the development of new therapeutic modalities. We thank all the participants for their contribution, especially those who came from outside Japan. We were able to make discussions as hot as the weather we had in Tokyo in July during the meeting.

